# Butterfly Eyespots: Their Potential Influence on Aesthetic Preferences and Conservation Attitudes

**DOI:** 10.1371/journal.pone.0141433

**Published:** 2015-11-06

**Authors:** Zoi Manesi, Paul A. M. Van Lange, Thomas V. Pollet

**Affiliations:** Social and Organizational Psychology, Department of Experimental and Applied Psychology, Faculty of Behavioural and Movement Sciences, VU Amsterdam, Amsterdam, The Netherlands; University of Stirling, UNITED KINGDOM

## Abstract

Research has shown that the mere presence of stimuli that resemble eyes is sufficient to attract attention, elicit aesthetic responses, and can even enhance prosocial behavior. However, it is less clear whether eye-like stimuli could also be used as a tool for nature conservation. Several animal species, including butterflies, develop eye-like markings that are known as eyespots. In the present research, we explored whether the mere display of eyespots on butterfly wings can enhance: (a) liking for a butterfly species, and (b) attitudes and behaviors towards conservation of a butterfly species. Four online experimental studies, involving 613 participants, demonstrated that eyespots significantly increased liking for a butterfly species. Furthermore, eyespots significantly increased positive attitudes towards conservation of a butterfly species (Studies 1, 2 and 4), whereas liking mediated the eyespot effect on conservation attitudes (Study 2). However, we also found some mixed evidence for an association between eyespots and actual conservation behavior (Studies 3 and 4). Overall, these findings suggest that eyespots may increase liking for an animal and sensitize humans to conservation. We discuss possible implications for biodiversity conservation and future research directions.

## Introduction

The “Samurai Crab” (*Heikea japonica*), a species of crab found in the southern Inland Sea of Japan, has become famous for the conspicuous markings on its shell that strikingly resemble the face of a Japanese samurai warrior. Huxley [1] (see also [2]) was so intrigued by the resemblance that he even suggested that the crab’s appearance was shaped by artificial selection. According to Huxley’s hypothesis, local fishermen would throw back into the ocean any crab that looked like a face, increasing the probability to survive and propagate for those *Heikea japonica* whose resemblance was closer to a human face. Notwithstanding some skepticism towards this hypothesis [3], it seems that humans are attracted by the appearance of objects that resemble faces, such as clouds with face-like formations (a phenomenon known as face pareidolia, see, e.g., [4,5]).

A naturally occurring face-like pattern in the environment is *eyespots*. Numerous animal species, including butterflies, birds, mollusks and fishes, possess eye-like body marks which are commonly called eyespots [6,7]. Research has demonstrated that eyespots can serve a function in interspecific encounters, as anti-predator mechanism (deterring potential attacks by mimicking the eyes of predators own enemies, see [8,9]) but also in intraspecific encounters, as sexually selected ornaments (see research on *Lepidoptera*, e.g., [10,11]).

Considering that humans are likely to protect animal species that they find beautiful [12–15], even small animal features that elicit aesthetic appreciation are perhaps sufficient to enhance conservation efforts for a given animal species. If humans are indeed attracted by face-like patterns in the environment, such as crabs with face-like elements [1,2], then the mere presence of eyespots on an animal may enhance liking, conservation attitudes and behavior towards such animal.

### Responses to eyes and eye-like patterns

Psychological research demonstrates that humans show spontaneous preference for eyes and stimuli that resemble eyes [16,17]. Infants and adults preferentially orient towards configurations possessing eye-like attributes at the expense of scrambled versions of faces or blank, unpatterned stimuli [18–21]. According to studies on attention, even the presence of three high-contrast blobs in a triangular formation corresponding to the relative location of eyes and mouth are sufficient to attract visual fixation [22,23]. Attraction to configurations that fit with face geometry has been associated with human susceptibility to high-contrasted elements in the upper visual field (i.e., positive contrast polarity and top-heavy bias) [22,24,25].

This heightened interest towards minimal eye-like stimuli is expressed not only through increased attention but also through enhanced evaluations and preferences. Empirical evidence from marketing research suggest that products with minimal cues in the form of a face (e.g., a car with headlights made to appear as eyes) can enhance product liking [26–28]. Furthermore, research on environmental conservation shows that simply assigning face-like characteristics to images of the natural world (i.e., anthropomorphism of nature) can enhance positive attitudes and protection of nature [29–31]. For example, a poster displaying the Earth as a face was found to increase people’s connectedness to nature and their inclination to engage in conservation behavior [29]. Likewise, a campaign poster displaying a tree with face-like characteristics was found to increase donations for a tree-planting campaign [31]. Thus, face-like configurations appearing on objects or images related to nature seem to increase attention, liking and even inclination to engage in conservation behavior.

An entity with face-like characteristics not only attracts attention but it can also elicit a feeling of surveillance, since a face could presumably be “watching” the individual’s behavior. This notion is supported by research from social psychology suggesting that a feeling of surveillance elicited by a pair of schematic eyes can enhance socially desirable and prosocial acts (for reviews, see [32,33]). For instance, Rigdon, Ishii, Watabe and Kitayama [34] provided evidence that participants in a dictator game showed greater generosity when they were presented with eye-like configurations (i.e., three blobs in a triangular configuration corresponding to eyes and mouth). Likewise, other studies have shown that the mere presence of images of eyes or eye-like shapes can enhance various socially desirable acts, including cooperation, charitable giving, voting in elections, conformity with the law and the rules [35–47] (but see, [48–53]).

Evidently, people do not perceive eye-like shapes as a real observer who can form impressions and judge the behavior of the individual. However, such false cues to surveillance can nonetheless elicit behavioral responses similar to those evoked by an actual observer because they appear to stimulate concerns about one’s own reputation. For example, findings by Oda, Niwa, Honma and Hiraishi [38] demonstrate that prosocial responses to eye-like shapes are related to expectations for improved reputation in the eyes of a third party. The fact that an individual tends to act prosocially in the presence of eye-like shapes, which cannot spread reputational information, is consistent with the notion of the existence of involuntary eye-detection mechanisms.

Recent work proposes that the human brain responds automatically and involuntarily to eyes and faces, even if those stimuli are schematic configurations [54,55]. Neuroimaging data show that, although people may not consciously perceive such stimuli as face patterns, configurations with eye-like elements are sufficient to activate cortical areas typically associated with face processing, such as the fusiform face area (FFA) [56,57]. In an attempt to explain this effect, it has been argued that specialized mechanisms designed to respond to cues to social scrutiny occasionally misfire to stimuli that are non-relevant to social presence [58–60].

A potential function for such a mechanism is that in ancestral contexts, stimuli that resemble eyes were likely to belong to an in-group member and, therefore, detecting them and responding prosocially was fundamental for cooperative (mutualistic) social interactions [61]. Although in modern contexts the presence of eye-like stimuli is not always a valid cue that observation is taking place, people show remarkable susceptibility to such cues.

### Present research

Taken together, there is thus evidence to suggest that minimal cues that look like eyes can attract people’s attention and enhance liking and prosocial behavior. In the present research, we sought to explore whether the mere display of eye-like patterns in an animal species can increase liking and prosocial behavior towards that particular animal species. If humans are susceptible to eye-like patterns in the environment, then perhaps eyespot display in an animal species can enhance aesthetic appreciation, conservation attitudes and conservation behavior towards a given animal species. Furthermore, we sought to explore if liking for eyespots explains potential effects of eyespots on conservation attitudes and conservation behavior. To test those predictions, we selected one butterfly species that bears characteristic eyespot patterns: the tropical butterfly *Bicyclus anynana (Lepidoptera*: *Nymphalidae*), a widely used model species in evolutionary biology [62].

In four online experiments, we tested the following hypotheses. First, if eyespots are indeed aesthetically appealing, we should find that butterflies will be rated as more “beautiful” or “attractive” when they contain eyespots as compared to no patterns (controls; Studies 1, 2 and 3) or other conspicuous features (i.e., stripes; Study 4). Second, if eyespots can increase prosocial attitudes, then attitudes towards the protection and conservation of butterflies will be more positive when the butterflies contain eyespots as compared to no patterns (Studies 1, 2 and 3) or other conspicuous features (Study 4). Third, if eyespots can increase prosocial behavior, then inclination to engage in conservation behavior towards butterflies will be greater when the butterflies have eyespots as compared to no patterns (Study 3) or other conspicuous features (Study 4). Fourth, if preference for eye-like structures is a potential psychological mechanism driving conservation, then aesthetic preferences for eyespots should mediate the effect of eyespots on conservation (Study 2).

## Study 1

In Study 1, we evaluated aesthetic preferences and conservation attitudes towards butterflies with eyespot patterns as compared to butterflies with no patterns (controls). *B*. *anynana* butterflies have eyespots which vary naturally in conspicuousness depending on the side of the wings and seasonality [63]: in the dry season, the upper (dorsal) wing surface contains pronounced large eyespots whereas the lower (ventral) surface is more cryptic, with the eyespot markings absent or reduced. To explore preferences for eyespots, we tested whether the spotted upper wings (as compared to the spotless lower wings) elicit greater aesthetic appreciation and more positive conservation attitudes towards those butterflies.

### Materials and Methods

#### Ethics statement

Ethical approval for all four studies was obtained from the Ethics Review Board (VCWE, Faculty of Behavioural and Movement Sciences, VU Amsterdam). At the beginning of the online study, participants read the study information and consented to participation by pressing the button to start the survey.

#### Participants

We recruited 101 American participants (44 men, 34 women, 23 participants did not provide information on gender, age range from 18 to 65 years) through Amazon’s Mechanical Turk, a website targeting a nationwide participant pool for online data collection [64]. The study was conducted as a within-participants design, in which participants received both the eyespot and the control condition in randomized order.

#### Eyespot manipulation and cover story

Participants were asked to read a short passage describing the morphology, natural habitat and several threats (e.g., pesticides, habitat loss and degradation) of a butterfly species, named Mitchell’s Satyr. According to the cover story, Mitchell’s Satyr is divided into two differentiated subspecies, which were called “Butterfly A” and “Butterfly B”. After reading the story, five pairs of digital photograph images were displayed on (the right and left side of) participant’s computer screen. Each pair of images depicted a different butterfly viewed from the spotted, dorsal wing surface (eyespot condition) and the spotless, ventral wing surface (control condition), respectively. Depiction of the eyespot and control condition images on the right, left part of the computer screen was randomized and the names (i.e., Butterfly A, B) were randomized between eyespot and control images. The two images of each pair had been pre-rated as similar in other key-features (i.e., wing color, shape and size; unpublished data). The five different butterfly stimuli were selected through the digital encyclopedia of Afrotropical butterflies compiled by Mark C. Williams (http://atbutterflies.com/). Permission to use the digital butterfly images for the purposes of the current research and publication purposes has been granted by the owner of the image database, Mark C. Williams. The butterfly photos used in the study and a task view are given in S1 and S2 Appendixs.

#### Ten-item measure of aesthetic preferences and conservation attitudes

We assessed aesthetic responses and conservation attitudes through a simple-choice instrument (see S3 Appendix). Specifically, while the pairs of spotted and spotless butterflies were displayed on the monitor, participants were asked to complete a 10-item questionnaire. Each item required the respondent to express preferences among three available options: (a) “Butterfly A”, (b) “Butterfly B”, or (c) “Neutral/no preference”. Items represented three different measures: (i) Aesthetics measure (items 1 and 2), (ii) Perceived fitness measure (items 3 and 4), (iii) Conservation attitudes measure (items 5, 6, 7, 8, 9 and 10).

As regards the Aesthetics measure, participants were classified into four different groups depending on their choices. Specifically, participants that consistently selected spotted butterflies were classified as having an aesthetic preference for eyespots. Participants that consistently selected spotless butterflies were classified as having an aesthetic preference for controls. Participants that consistently selected the “Neutral/no preference” option were classified as having no preference. Participants that did not consistently select spotted, spotless or the “Neutral/no preference” option were classified as having mixed preferences. The same classification was used for the Perceived fitness measure. Conservation attitudes were indicated by the tendency to make four consistent choices (i.e., spotted butterfly, spotless butterfly or neutral) when filling out the Conservation attitudes measure. For evaluating Conservation attitudes, we used the same methodology used in research using forced-choice methodology and decomposed economic games (see e.g., [65]). We also measured average preference for protecting spotted butterflies.

#### Covariates and statistical analyses

We tested for effects with and without each of the following variables: general environmental attitudes, involvement in pro-environmental activities, and some demographics (e.g., gender, age). General environmental attitudes were assessed through the 15-item revised New Environmental Paradigm (NEP-R, [66]). Items were rated on a 5-point Likert scale (*1 = Strongly Disagree* to *5 = Strongly Agree*) and an example item is: *“When humans interfere with nature*, *it often produces disastrous consequences”*. After reverse scoring, higher scores indicated greater concern for the environment (Cronbach’s α = .87). Participants were predominantly neutral on their levels of concern for the environment (*M* = 3.51, *SD* = .64). Involvement in pro-environmental activities (PEA) was measured through a purpose-built instrument, which contained four items (each scored on a 5-point Likert scale ranging from *1 = Never* to *5 = Several times a month*) that assessed the extent to which participants have taken four different actions in the last 5 years (i.e., sign a petition about an environmental issue, volunteer time or donate money to an environmental organization, take part in a protest about an environmental issue, take action to protect a butterfly species). The four items were averaged into a reliable scale (Cronbach’s α = .83) and the vast majority of participants reported little or no involvement in actions for the protection of the environment or butterflies (*M* = 1.40, *SD* = .65).

We used chi-square tests to assess the role of eyespots in predicting aesthetic preferences and conservation attitudes towards butterflies. Ordinal correlations (Spearman rho [67]) were used to examine if preferences for the spotted butterflies are related to demographic variables, NEP-R or PEA. All analyses were conducted in SPSS 20.0 [68]. Data can be found in S1 Data.

### Results

#### Main analyses

**Fig 1:**
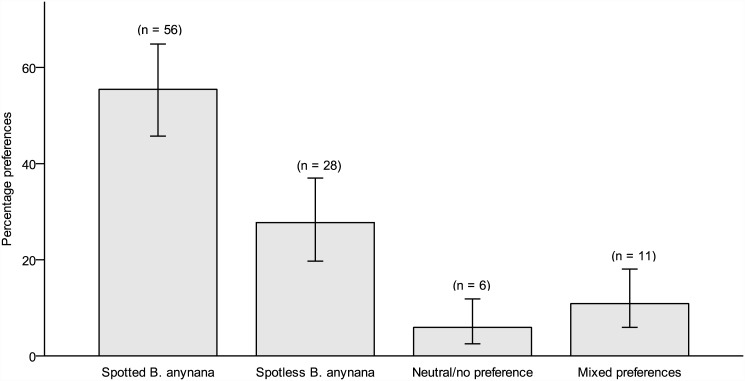
Aesthetic preferences for eyespots (Study 1). The mean (± S.E.) percentage of aesthetic preferences for spotted versus spotless *B*. *anynana* butterflies in Study 1.

A chi-square test on the aesthetics measure showed that there was significant variation in aesthetic preferences (χ^2^(3) = 60.47, *p*< .001), with more participants preferring spotted butterflies (*n* = 56) compared to those preferring spotless ones (*n* = 28) and those having neutral preferences (*n* = 6) or mixed preferences (*n* = 11). The results are shown in Fig 1. Another chi-square test on the perceived fitness measure indicated significant variation in fitness preferences (χ^2^(3) = 33.22, *p*< .001), with more participants attributing greater fitness to spotted butterflies (*n* = 46) over spotless ones (*n* = 18), or expressing neutral (*n* = 7) or mixed preferences (*n* = 30). These results suggest that the mere presence of eyespots can increase perceived attractiveness and fitness of a butterfly.

Next, we aimed to determine whether there is a statistically significant difference in conservation attitudes towards spotted versus spotless butterflies. A chi-square test on the conservation attitudes measure showed that there was significant variation in conservation attitudes (χ^2^(3) = 58.41, *p*< .001), with participants showing increased preferences for conservation of spotted butterflies. Specifically, the vast majority of participants (*n* = 58) tended to select spotted butterflies, whereas fewer participants tended to prefer spotless butterflies (*n* = 18), express neutral preferences (*n* = 16) or mixed preferences for conservation (*n* = 9). Additionally, a one-sample t-test showed that mean preference for conservation of spotted butterflies is significantly greater (*M* = 3.53, *SD* = 2.39) than the expected mean preference (i.e., selecting spotted butterflies in three out of six items), t(100) = 2.24, *p* = .027. These results suggest that the mere presence of eyespots can elicit greater support (and willingness to get involved in) conservation efforts targeted towards a butterfly.

#### Covariates

Finally, as indicated by Spearman’s rank order correlation coefficient, there was no significant relationship between any of the control variables (NEP-R scale, purpose-made PEA scale, gender, age) and aesthetic preferences or conservation attitudes (*Spearman’s rho’s*< .20, *p’*s> .08). There was only a weak positive relationship between gender and perceived fitness (*Spearman’s rho* = .25, *p* = .025). Because we had no specific prediction regarding perceived fitness, items measuring perceived fitness were omitted from the following studies.

## Study 2

In Study 2 we aimed to replicate the effect of eyespots on aesthetic preferences and conservation attitudes towards butterflies by using a between-participants design. Furthermore, we sought to explore the mediating role of aesthetics for the relationship between eyespots and attitudes for butterflies’ conservation. Do people support the protection of spotted butterflies (rather than spotless ones) because they perceive eyespots as a particularly beautiful feature?

### Materials and Methods

#### Participants

An Amazon’s Mechanical Turk survey was completed by 208 US participants (90 men, 118 women, age range from 18 to 65 years). After participants followed the hyperlink on MTurk to the online study, they were randomly assigned to the eyespot (*n* = 94) or control condition (*n* = 114). Cover story and experimental manipulation (i.e., butterfly stimuli) were identical to those used in Study 1 (see S1 Appendix), with one important difference: participants viewed either spotted butterflies (eyespot condition) or spotless butterflies (control condition) while reading the story and responding to items.

#### Measure of aesthetic preferences and measure of conservation attitudes

We used a single-item measure of aesthetic preferences: participants rated on a 5-point scale (*1 = Not at All* to *5 = To a Very Great Extent*) how beautiful or aesthetically pleasing they perceived the presented butterfly stimuli. Higher scores indicated greater aesthetic preference. Next, we assessed conservation attitudes, using two different items. The first item measured concern for butterfly protection *(“To what extent do you think that the depicted butterfly should be protected and maintained*?*”*). The item was rated on a 5-point scale (*1 = Not at All* to *5 = To a Very Great Extent*) and higher scores indicated higher concern for butterfly protection. The second item assessed support for conservation actions towards butterflies: participants were asked to skim through a list of actions aimed at the conservation of the depicted butterflies and to select as many of the enlisted actions they believed that should be taken by governmental agencies, social media and individuals. Example actions included civil penalties for illegal collecting or habitat disturbance, investment of resources to butterfly conservation programs, and raising public awareness through social media. The greater the number of conservation actions selected, the greater the support for conservation actions towards butterflies.

#### Covariates and statistical analyses

We performed tests with and without each of the following variables: the 15-item NEP-R scale [66] (Cronbach’s α = .86, *M* = 3.55, *SD* = .58), the 4-item purpose-built PEA measure (Cronbach’s α = .72, *M* = 1.50, *SD* = .60) and some demographic variables (e.g., gender, age). The Generalized linear model (GzLM) was preferred over the classical General Linear Model (GLM) because it offers increased flexibility in modeling dependent variables [69–71]. Two alternative models, the Linear model and the Poisson model, were tested. Given that our data can be seen as count data, these Poisson models were corrected for over- or underdispersion [70]. Model selection was based on the Akaike Information Criterion (AIC; [72]) values, with lower AIC values indicating a better model fit to the data [73]. After comparing models by AIC differences (ΔAIC), the model that proved to be a better fit to the data was selected, although both models yielded the same conclusions. Analyses were conducted in SPSS 20.0 [68]. The confidence intervals and *p*-values we report are 95% confidence intervals as based on bias-corrected accelerated bootstraps of 1,000 samples each [74,75]. These bootstrapped confidence intervals do not have parametric assumptions. Mediation was tested via the SPSS macro PROCESS for a bootstrapped cross-product test with 10,000 replicates [76]. Data can be found in S1 Data.

### Results

#### Main analyses

First, we performed a GzLM for aesthetic preferences. A linear model proved a better fit than a Poisson model (ΔAIC = 69.58 in favor of the Linear model). The GzLM for aesthetic preferences demonstrates that a model that included a main effect for eyespots fits the data significantly better than the intercept-only model (*B(eyespots)* = .67 (95% CI: 0.32–0.99), Wald χ²(1) = 17.83, *p* = .001). Participants who viewed the spotted butterfly tended to rate the butterfly as more beautiful (*M* = 3.77, *SD* = 1.04, *Grouped Mdn* = 3.82) than participants who viewed the spotless butterfly (*M* = 3.10, *SD* = 1.21, *Grouped Mdn* = 3.05, *Cohen’s d* = .59). Thus, the presence of eyespots was found to increase perceived attractiveness of a butterfly.

**Fig 2:**
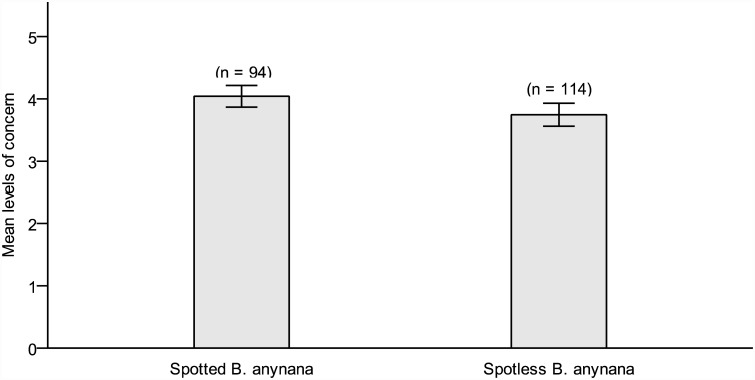
Levels of concern for butterfly conservation (Study 2). Boxplots depicting levels of concern (± S.E.) for conservation of spotted versus spotless *B*. *anynana* butterflies in Study 2. Levels of concern are measured on a 5-point Likert scale.

Next, we performed two GzLMs to explore the eyespot effect on conservation attitudes towards butterflies. The first GzLM focused on concern for butterfly protection. A linear model proved a better fit than a Poisson model (ΔAIC = 155.68 in favor of the Linear model). The GzLM for concern for butterfly protection, including eyespots as a main effect, was found to fit the data significantly better than the intercept-only model (*B(eyespots)* = .30, (95% CI, 0.07–0.54), Wald χ²(1) = 5.22, *p* = .021). In comparison to the spotless butterfly, the log likelihood of expressing concern for the protection of the spotted butterfly was significantly higher. As shown in Fig 2, the mean value of the eyespot group (*M* = 4.04, *SD* = .85, *Grouped Mdn* = 4.12) was slightly, yet significantly, higher compared to that of the control group (*M* = 3.75, *SD* = .99, *Grouped Mdn* = 3.80, *Cohen’s d* = .31).

The second GzLM focused on support for conservation acts. A Poisson model proved a better fit than a Linear fit (ΔAIC = 101.98 in favor of the Poisson model). The GzLM for support for conservation actions towards butterflies, including eyespots as a main effect, fits the data significantly better than the intercept-only model (*B(eyespots)* = .21 (95% CI, 0.07–0.34), Wald χ²(1) = 7.84, *p* = .003). In comparison to the control condition, the log-likelihood of supporting conservation actions towards butterflies was greater in the eyespot condition. On average, participants in the eyespot group (*M* = 3.45, *SD* = 1.56, *Grouped Mdn* = 3.43) tended to support significantly higher number of actions for butterfly protection compared to participants in the control group (*M* = 2.80, *SD* = 1.54, *Grouped Mdn* = 2.78, *Cohen’s d* = .42). Even though *Cohen’s d* is imperfect for Poisson models, here we use this effect size index for reasons of comparison. This result suggests that the mere presence of eyespots can yield more positive attitudes towards the protection of a butterfly.

The main effect of eyespots on each of the three outcome variables remained significant (all *p’*s< .032) after controlling for covariates (i.e., NEP-R scale, PEA purpose-made scale, gender and age). Thus, the presence of eyespots significantly increased perceived attractiveness of butterflies and led to more positive attitudes towards conservation of butterflies.

#### Mediation analyses

**Fig 3:**
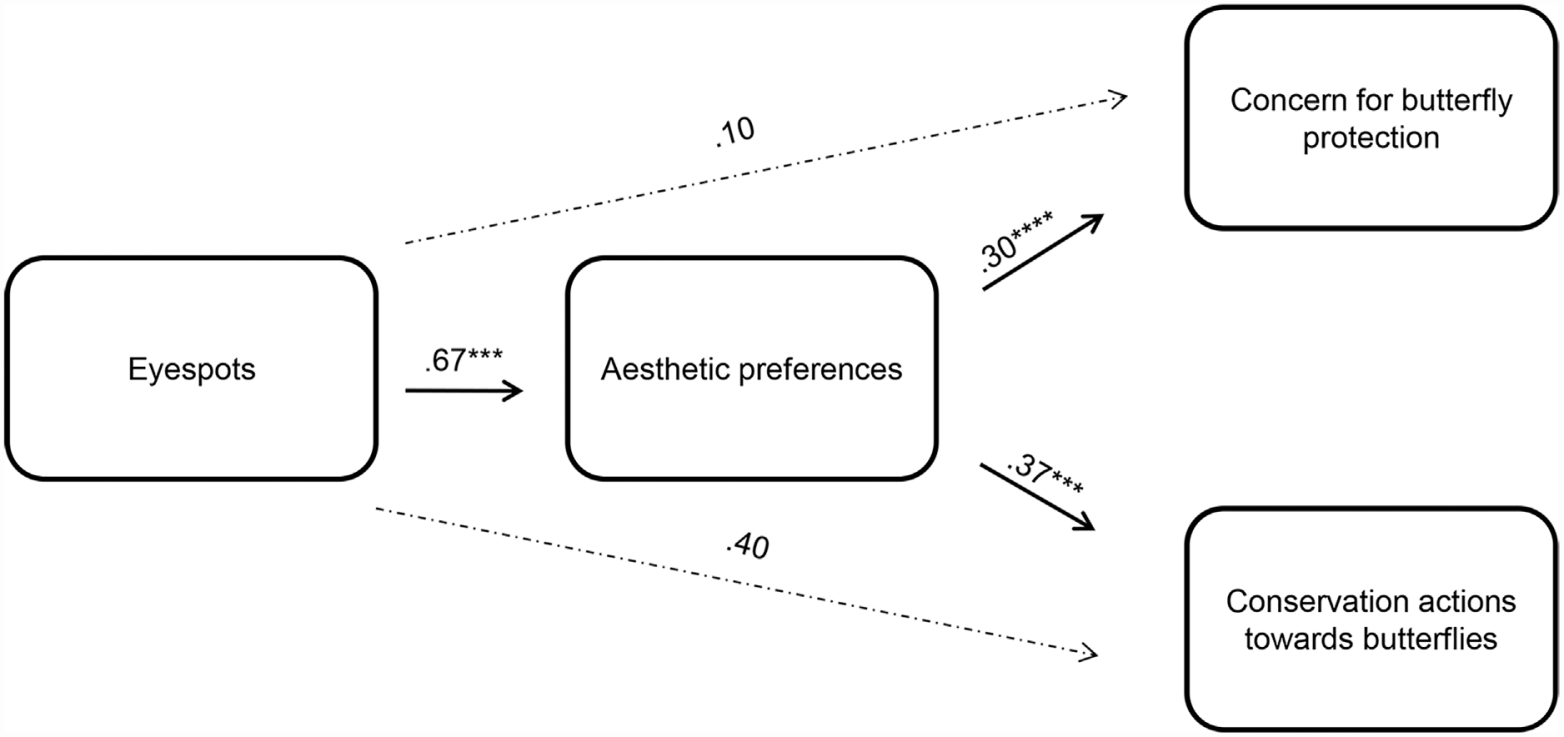
Aesthetics as a mediator of the relationship between eyespots and butterfly conservation attitudes (Study 2). Schematic model depicting the mediating role of aesthetic preferences in: (a) the relationship between eyespots and concern for conservation of *B*. *anynana* butterflies, and (b) the relationship between eyespots and support for conservation actions towards *B*. *anynana* butterflies. *Note*. ****p< .00001, ***p≤ .001. Entries are unstandardized regression coefficients and *n* = 208.

We explored the potential mediating role of aesthetic preferences in the relationship between eyespots and conservation attitudes towards butterflies, using two statistical approaches. First, we focused on the levels of concern for butterfly protection (DV2). Regression analyses demonstrated a statistical significance for both the a-path (eyespot effect on aesthetic preferences, *B* = .67, *SE*(B) = .16, *t*(206) = 4.22, *p* = .001, 95% CI, 0.34–0.95) and the b-path (eyespot effect on concern for butterfly protection, *B* = .30, *SE*(B) = .13, *t*(206) = 2.28, *p* = .027, 95% CI, 0.05–0.53). Based on this result, a mediation analysis was conducted (see [76] using 10,000 bootstrap replicates). As shown in Fig 3, data from the mediation analysis support the mediating role of aesthetic preferences in the relationship between eyespots and concern for butterfly protection (*B* = .30, *SE*(B) = .05, *t*(205) = 5.60, *p*< .00001, 95% CI, 0.19–0.40). After controlling for aesthetic preferences, the effect of eyespots on the levels of concern for butterfly protection became non-significant (*B* = .10, *SE*(B) = .13, *t*(205) = .77, *p* = .445, 95% CI, -0.15–0.35), which suggests that there is a full mediation of the effect.

Second, we focused on the number of actions for butterfly protection (DV3): we explored the potential mediating effect of aesthetic preferences on the relationship between eyespots and conservation actions towards butterflies. Both the a-path (see above) and the b-path (*B* = .65, *SE*(B) = .22, *t*(206) = 3.01, *p* = .003, 95% CI, 0.18–1.05) were statistically significant. As shown in Fig 3, data from a mediation analysis using 10,000 bootstrap replicates confirmed the mediating role of aesthetic preferences in the relationship between eyespots and conservation actions towards butterflies (*B* = .37, *SE*(B) = .09, *t*(205) = 4.02, *p* = .0001, 95% CI, 0.19–0.55). After controlling for aesthetic preferences, the effect of eyespots on conservation actions towards butterflies becomes non-significant (*B* = .40, *SE*(B) = .22, *t*(205) = 1.86, *p* = .065, 95% CI, -0.03–0.83), which indicates that there is again a full mediation of the effect.

## Study 3

Study 3 had two main aims. The first aim was to replicate the between-participants effect of eyespots on aesthetic preferences using a continuous-rating method (i.e., a slider on a continuous scale) rather than a five-point scale. Responses in the slider measure can yield a full ranking of preferences over butterfly eyespots providing a clearer picture of participants’ aesthetic preferences. The second aim was to offer a deeper understanding of the eyespot effect by testing whether eyespots can affect actual behavior aimed at conservation and protection of butterflies. We measured two different behaviors: (a) inclination to get informed about butterfly conservation efforts, and (b) willingness to make a donation to support a butterfly conservation program.

### Materials and Methods

#### Participants

Amazon’s Mechanical Turk was used to recruit 203 US participants (99 men, 101 women, 3 participants did not provide information on gender, age range from 18 to 65 years), who were randomly assigned to the eyespot (*n* = 102) or control condition (*n* = 101). The eyespot manipulation and cover story were identical to those of Study 2.

#### Measure of aesthetic preferences and behavioral measures of conservation

Participants rated on a continuous scale (*0* = *Not at All* to *100 = To a Very Great Extent*) the extent to which they perceived the depicted butterflies as beautiful or aesthetically pleasing, with higher scores indicating greater aesthetic preference. As regards behavioral measure 1, participants were provided with a simple binary choice (i.e., “watch” or “not watch”) and were asked to decide whether they wished to spend few minutes watching an informative video about conservation efforts relevant to the endangered butterfly species of the survey. Greater inclination to watch the video indicated greater willingness to spend time learning about butterfly conservation efforts. Behavioral measure 2 assessed donating behavior for protection of the endangered butterfly of the survey: participants were provided with a simple binary-choice (“donate money” or “not donate money”) and were asked to indicate whether they wished to donate their payment for participation in the survey ($0.25) to a conservation program targeting the endangered butterfly. While participants were making their decisions in these two behavioral tasks, they were exposed either to a spotted butterfly or to a spotless butterfly.

#### Covariates and statistical analyses

We performed tests with and without each of the following variables: the 15-item NEP-R scale [66] (Cronbach’s α = .87, *M* = 3.57, *SD* = .61), the 4-item purpose-built PEA measure (Cronbach’s α = .77, *M* = 1.55, *SD* = .63) and demographic variables (gender, age). We used a GzLM in SPSS 20.0 to explore the eyespot effect on aesthetic preferences. A Poisson model, corrected for over/underdispersion, was selected because it yielded a better fit than the Linear (identity link) model (ΔAIC = 144420). Model selection was based on Akaike Information Criterion (AIC; [72]) values, with low AIC values indicating better model fit to the data). Next, we used chi-square tests to examine the effect of eyespots on the two behavioral measures of conservation. Data can be found in S1 Data.

### Results

#### Main analyses

A GzLM for aesthetic preferences including eyespots as a main effect fitted the data significantly better than the intercept-only model (*B(eyespots)* = .21, *SE*(B) = .07, Wald χ²(1) = 7.62, *p* = .007, 95% CI, 0.05–0.35). Compared to the spotless butterfly, the likelihood of rating the spotted butterfly as beautiful or aesthetically pleasing was higher. On a 100-point slider scale, the spotted butterfly received aesthetic ratings above average levels (*M* = 59.55, *SD* = 26.75, *Grouped Mdn* = 60.86) whereas the spotless butterfly received aesthetic ratings below average (*M* = 48.53, *SD* = 27.56, *Grouped Mdn* = 41, *Cohen’s D* = .41). The main effect of eyespots on aesthetic preferences remained highly significant (all *p’s*< .01) after controlling for covariates (i.e., NEP-R scale, PEA purpose-made scale, gender and age).

A chi-square test on behavioral measure 1 (i.e., inclination to get informed about butterfly conservation) showed that there was no significant variation in inclination to get informed about butterfly conservation (χ^2^(1) = 0.17, *p* = .895), with comparably low numbers of participants exposed to spotted butterflies (*n* = 28) and spotless butterflies (*n* = 28) being willing to get informed. Next, a chi-square test on behavioral measure 2 (i.e., donating behavior) showed that there was no significant variation in donating behavior (χ^2^(1) = .006, *p* = .941), with comparably low numbers of participants exposed to spotted butterflies (*n* = 11) and spotless butterflies (*n* = 11) being willing to donate their participation fee to save butterflies. These results thus suggest that although eyespots are effective enough to influence aesthetic judgments they are perhaps less powerful in eliciting certain behaviors for the protection of butterflies.

## Study 4

Studies 1, 2 and 3 provided initial support for the hypothesis that eyespots elicit aesthetic responses and positive attitudes towards the protection of a butterfly species. However, an alternative interpretation of those data could be that any conspicuous pattern on butterfly wings could affect responses and attitudes towards butterflies. Therefore, in Study 4 we included another control condition; that is images of butterflies with conspicuous stripes (instead of eyespots). Furthermore, one could claim that participants in Studies 1, 2 and 3 preferred spotted (over spotless) butterflies because other minimal morphological differences between upper and lower wing surfaces may render upper wings more attractive than lower wings. Thus, in Study 4 we displayed only images depicting the upper wing surface of butterflies. Also, we measured conservation behavior in a slightly different way; instead of donating the participation fee (Study 3), participants were asked whether they wished to donate a further sum to a butterfly conservation charity.

### Materials and Methods

#### Participants

We recruited participants through CrowdFlower, a crowdsourcing system that delegates work to various other platforms including Amazon’s Mechanical Turk. The sample consisted of 101 US participants (18 men, 81 women, 2 participants did not provide information on gender, age range from 18 to 66 years). The experiment was run as a within-participants design, in which participants were subject to three randomized conditions: eyespots, stripes, and no eyespots. The latter two conditions served as two distinct baselines (control conditions) for assessing the effects of eyespots.

#### Eyespot manipulation

After reading the cover story used in Study 1, participants sequentially viewed five triads of butterflies. Each triad included digitally altered images of a *B*. *anynana* butterfly (dorsal view, see S4 Appendix). First, the image of the spotted butterfly was manipulated such that the eyespot area remained intact but any other markings (e.g., line patterns or other smaller spots on wings) were digitally removed. Removing other minimal markings could allow us to test whether responses to butterflies are only related to eyespots. Second, the image of the spotless butterfly was manipulated such that the eyespots and any other minimal markings were digitally removed. Third, the image of the striped butterfly was manipulated such that stripes were displayed on the upper butterfly wings whereas the eyespot area and any other markings were digitally removed. We generated stripes that matched the pixel size and color tones of the eyespots. Digital alterations and stimuli generations were performed with Adobe Photoshop CS5.1. Each stimulus within the triad was presented on the right, center or left side of the computer screen. As in Study 1, the location in which each of the three butterfly types appeared was randomized across participants and across questions. All images can be found in S4 Appendix.

#### Measure of aesthetic preferences and behavioral measures of conservation

Questions on aesthetic preferences and conservation attitudes were identical to those used in Study 1. Each item required the respondent to express preferences among three available options: spotted butterfly, striped butterfly or control butterfly. As regards actual conservation behavior, instead of donating their participation fee (see Study 3), participants were given the option to donate a further monetary sum to a butterfly conservation charity, namely “Butterfly Conservation” (butterfly-conservation.org). Specifically, participants were presented with three butterfly stimuli (i.e., spotted butterfly, spotless butterfly, striped butterfly) and a no-donation option. They were informed that they could donate any sum they wished to save one of the three butterflies (or none of them). According to the cover story, the butterfly stimuli presented on the screen had characteristics similar to those found in actual butterflies (i.e., in the website participants could indeed select from a variety of butterflies to support). Participants who selected to provide monetary resources to one of the butterfly stimuli, were re-directed to the website of “Butterfly Conservation”.

In Study 4, we did not administer measures of environmental attitudes and involvement in pro-environmental activities, since such variables had no significant effect in Studies 1, 2 and 3. Statistical analyses were identical to those used in Study 1. Data can be found in S2 Data.

### Results

#### Main analyses

**Fig 4:**
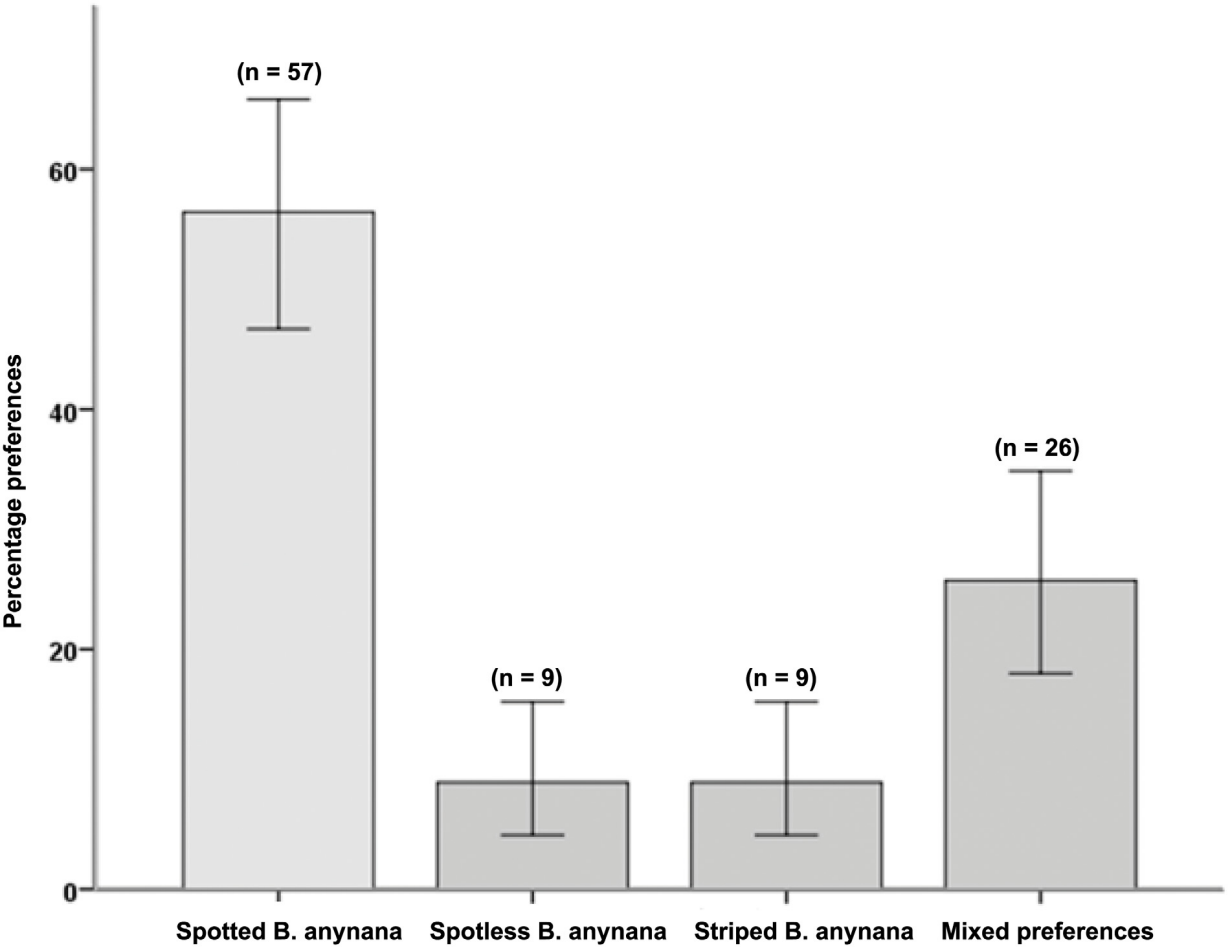
Aesthetic preferences for eyespots (Study 4). The mean (± S.E.) percentage of aesthetic preferences for spotted as compared to spotless or striped *B*. *anynana* butterflies in Study 4.

A chi-square test on the two-item measure of aesthetics showed that there was significant variation in aesthetic preferences (χ^2^(3) = 60.86, *p*< .001), with more participants preferring spotted butterflies (*n* = 57) compared to those preferring spotless butterflies (*n* = 9), those preferring striped butterflies (*n* = 9) or those having mixed preferences (*n* = 26). This result provides further evidence that participants perceive eyespots to be a more attractive feature than another conspicuous pattern element (stripes) or no pattern element (see Fig 4).

**Fig 5:**
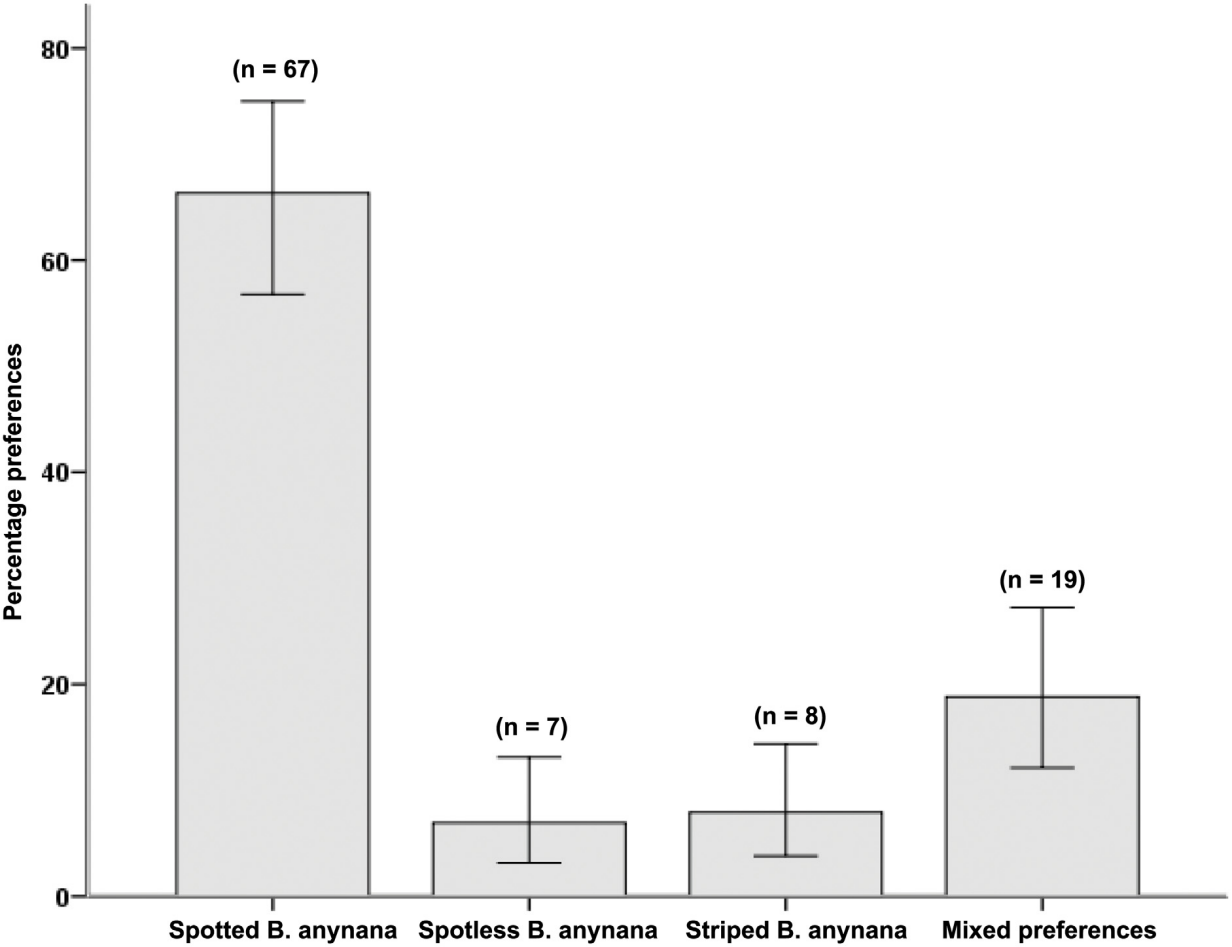
Attitudes towards butterfly conservation (Study 4). The mean (± S.E.) percentage of preferences regarding conservation of spotted as compared to spotless or striped *B*. *anynana* butterflies in Study 4.

Next, a chi-square test on the six-item measure of conservation attitudes showed that there was significant variation in conservation attitudes (χ^2^(3) = 95.55, *p*< .001), with participants showing more positive conservation attitudes towards spotted butterflies. The majority of participants (*n* = 67) preferred to protect spotted butterflies, and significantly fewer participants preferred to protect spotless butterflies (*n* = 7), striped butterflies (*n* = 8), or expressed mixed preferences for the butterflies (*n* = 19). Additionally, a one-sample t-test showed that mean preference for conservation of spotted butterflies is significantly greater (*M* = 4.08, *SD* = 2.40) than the expected mean preference (i.e., selecting spotted butterflies in two out of six items), *t*(100) = 8.73, *p* = .001. Thus, participants were positively predisposed towards the protection of butterflies with eyespots. The results are shown in Fig 5.

Finally, a chi-square test on the behavioral measure of conservation, showed that across conditions, a significant majority expressed a preference to make no donation for saving butterflies (χ^2^(3) = 139.75, *p*< .001). However, among participants who decided to donate a further sum to protect butterflies, there was significantly greater preference to donate for spotted butterflies (χ^2^(2) = 26.39, *p*< .001). Specifically, more participants wished to donate to save spotted butterflies (*n* = 21) as compared to spotless butterflies (*n* = 2) or striped butterflies (*n* = 3).

In sum, the presence of eyespots significantly increased perceived attractiveness of butterflies and led to more positive conservation attitudes towards butterflies. Furthermore, for participants who decided to engage in conservation behavior, the likelihood of donating to save butterflies was greater towards butterflies with eyespots.

## Discussion

Findings from four empirical studies provided support for most of our hypotheses. All four studies demonstrated that the presence of eyespots on butterfly wings could enhance liking for a butterfly. Furthermore, Studies 1, 2 and 4 provided good evidence that the mere display of eyespots can elicit positive attitudes towards conservation of a butterfly species. As regards conservation behavior, results were mixed: the presence of eyespots enhanced behavior towards conservation of butterflies in Study 4 but not in Study 3.

The present research reveals that a majority of people has a clear preference for eyespots. This finding agrees with past research showing that face-like configurations and eye-like shapes attract attention and affect liking (see, e.g., [21,22,26]). In four independent studies, we show that the mere presence of eyespots on butterflies is sufficient to enhance aesthetic responses, as expressed by judgments of attractiveness and explicit preferences. This suggests that the effect of eye-like stimuli on preferences is not restricted solely to schematic shapes or consumer products but it is also evident when humans evaluate another species. Although, it has long been known that physical traits (e.g., color, charisma) affect people’s aesthetic preferences in animal species [12,13], here we show that even minor cues (such as eyespots) can be efficient in influencing human aesthetic preferences. Although we cannot yet formulate conclusions on the generalizability of the eyespot effect in other animal species, this research provides initial evidence that features resembling socially relevant cues, such as eyespots, can make people discriminate among similar organisms.

The fact that aesthetic ratings were higher for butterflies with eyespots as compared to those with stripes (Study 4) further strengthens our conjecture that it is eyespots in particular that heighten aesthetic appeal rather than any conspicuous pattern. Since morphological characteristics (e.g., color tones, size and symmetry) of the two types of stimuli (i.e., eyespots and stripes) were matched, this finding indicates that aesthetic preferences were not merely driven by chromatic contrast but rather by eye-like stimuli. It was also interesting to see that, aesthetic ratings for butterflies bearing eyespots were even higher when participants were also presented with other kinds of butterflies (i.e., butterflies with no patterns or stripes, Studies 1 and 4), as compared to when there were no reference stimuli (Studies 2 and 3).

Furthermore, by drawing attention to the role of eyespots in conservation attitudes, the present research complements and extends previous work in the area of social and environmental psychology. Past studies have shown that anthropomorphism of nature (e.g., giving face-like characteristics to images of nature) fosters environmental concern and environmentally friendly attitudes [29–31]. In three of our studies, we demonstrated that the mere presence of eyespots can increase positive attitudes towards conservation of butterflies. Participants expressed greater concerns about extinction risk and higher support for conservation efforts targeting butterflies with eyespots as compared to butterflies with no patterns (Studies 1, 2 and 4), or stripes (Study 4).

Importantly, these findings also uncovered that aesthetic preferences mediated the effect of eyespots on conservation attitudes. Although past studies have linked such attitudinal changes to factors like connectedness to nature or heightened feelings of guilt [29,31], our finding suggests that preference for face-like patterns may be an underlying psychological mechanism; eyespots influence aesthetic preferences, which in turn facilitate prosocial attitudes towards an animal species. These findings, thus, support the notion that humans are remarkably susceptible to “faceness” and they have an innate tendency to be attracted and respond prosocially to cues that resemble a face (or just eyes, see, e.g., [34,35,55,77]). Enhanced liking for eyespots appears to be a powerful drive influencing conservation attitudes.

Although our measures reveal a strong link between aesthetic preferences and conservation attitudes, we cannot exclude the possibility that other processes may underlie conservation. For instance, studies on the “eye-images effect” propose that elevated interest and prosociality in the presence of eye-like stimuli stems from a predisposition to respond to cues indicating surveillance (e.g., [44,58]). Over the course of human evolution, paying attention to cues to social surveillance and adjusting one’s behavior in a socially desirable manner could have facilitated profitable social relationships (see, e.g., [78–80]). Contrariwise, failure to respond prosocially in the presence of indicators of social attention could jeopardize one’s own reputation and result in social sanctions [81–83].

As with eye-like stimuli that have been used in past studies (see, e.g., [34,35]), eyespots are not realistic and, thus, are unlikely to be perceived as a real observer. However, the fact that participants showed higher levels of prosocial attitudes towards butterflies with eyespots (as compared to butterflies with no patterns or stripes) could suggest the existence of an involuntary eye-detection mechanism [58,60]. Although eyespots are only minimally similar to eyes, they may be sufficient in activating an involuntary cognitive mechanism for detecting social gaze. Future research is required to examine whether a sense of surveillance can explain the eyespot effect on conservation attitudes. We cannot exclude the possibility that other processes may guide aesthetic judgments and conservation attitudes. For example, participants in our studies may have relied on heuristic processes such as positive or negative feelings (see, e.g., “affect heuristic”). Such feelings can provide valuable information that help in simplifying judgments and decisions. Future studies should consider the use of affective evaluations.

Our data suggest that eyespots not only enhance conservation attitudes but, under certain circumstances, they may also affect preferences in conservation behavior. Eyespots did not increase inclination to receive information or donate one’s participation fee for butterfly conservation (Study 3), but they affected the inclination to donate a further amount of money to save butterflies (Study 4). Participants who decided to donate to a butterfly charity were significantly more likely to donate for saving butterflies with eyespots rather than those with stripes or no patterns. Restated, individuals who engaged in charitable behavior showed bias in supporting butterflies with eyespots. This possibility, raised by the present findings, suggests that eyespots may not be a sufficient social nudge to increase overall conservation behavior (i.e., a minority of participants actually donated to save butterflies), but they can make charitable givers prioritize saving butterflies with eyespots over others.

Additional research is required to further investigate the effect of eyespots on various kinds of conservation behavior. It needs to be underlined that the present paper is one of the first, to our knowledge, that utilized specific behavioral measures of conservation. Past research from environmental psychology has mainly focused on conservation attitudes or expressed inclination to engage in environmentally friendly behaviors rather than actual conservation acts (see, e.g., [29,31,84,85]).

The above evidence could have potentially important implications for biodiversity conservation and ecology. A robust effect of eyespots on conservation attitudes would underline the necessity to pay attention to the important role of relatively subtle features of organisms in sensitizing humans to conservation. Considering that invertebrates have generally received little public attention, understanding the appeal of minimal, yet powerful, features can help identify effective invertebrate species for conservation flagships. For instance, if eyespot color patterns can confer aesthetic charisma to invertebrates, then the use of endangered spotted butterflies, like Mitchell’s Satyr or Saint Francis’s Satyr (see FWS, 2015), in flagship campaigns could be an instrument in conservation. This could galvanize public support not only for those specific species but also for the preservation of their natural habitat. Whether positive attitudes towards conservation of spotted animal species translates into prioritizing funding to save those animals remains to be demonstrated, however.

A methodological limitation of the present research is the lack of other species, apart from butterflies, that could be used in the assessment of the eyespot effect. One could argue that this may threaten the generalizability of our results to other species with face-like patterns. In future research we aim to test whether similar eyespot effects apply to a range of other animals. Another limitation is that we compared eyespots with artificial stimuli (i.e., stripes), rather than other naturally occurring patterns. It is possible that participants doubted whether the striped butterfly stimuli were real. Although this possibility cannot be ruled out, it is noteworthy that no participant indicated suspicion regarding the artificial stimuli. Furthermore, conclusions regarding the superiority of eyespots over other conspicuous features in predicting preferences are limited to the patterns that we used in the present research. Further research is needed to clarify whether eyespots are a stronger predictor of aesthetics and conservation as compared to various other naturally occurring patterns. Future exploration of the eyespot effect could involve visual attention tracking, which could complement self-report measures for preferences.

## Conclusion

The present research contributes to a growing body of knowledge uncovering the effects of eye-like shapes on judgments of attractiveness, preferences and prosociality. The broader message from four studies reported here is that the presence of eyespots on butterflies can promote their perceived attractiveness, liking and attitudes regarding their conservation. We also provided evidence that these findings were not observed when eyespots were replaced with stripes, patterns that do not resemble eyes. This finding suggests that minimal social cues (in the form of eyes) may be a useful strategy for drawing attention to threatened animal species and their conservation. Future research and campaigns for animal protection could demonstrate the necessity of considering minimal animal characteristics, such as eyespots.

## Supporting Information

S1 Appendix Butterfly stimuli (Studies 1, 2 and 3). (PDF)

S2 Appendix Task view (Study 1). (TIF)

S3 Appendix Ten-item measure of aesthetic preferences and conservation attitudes (Studies 1 and 4). (PDF)

S4 Appendix Butterfly stimuli (Study 4). (ZIP)

S1 Data Data sets (Studies 1, 2 and 3). (ZIP)

S2 Data Data set (Study 4). (ZIP)
