# Supplementary material for: Butterfly Eyespots: Their Potential Influence on Aesthetic Preferences and Conservation Attitudes
Source: PLoS One. 2015 Nov 6;10(11):e0141433. doi: 10.1371/journal.pone.0141433 (PMC4636354; doi:10.1371/journal.pone.0141433)

Butterfly stimuli (Studies 1, 2 and 3)

Spotted and spotless *B. anynana* butterflies used in a within-participants design (Study 1, randomized position) and in a between-participants design (Studies 2 and 3).

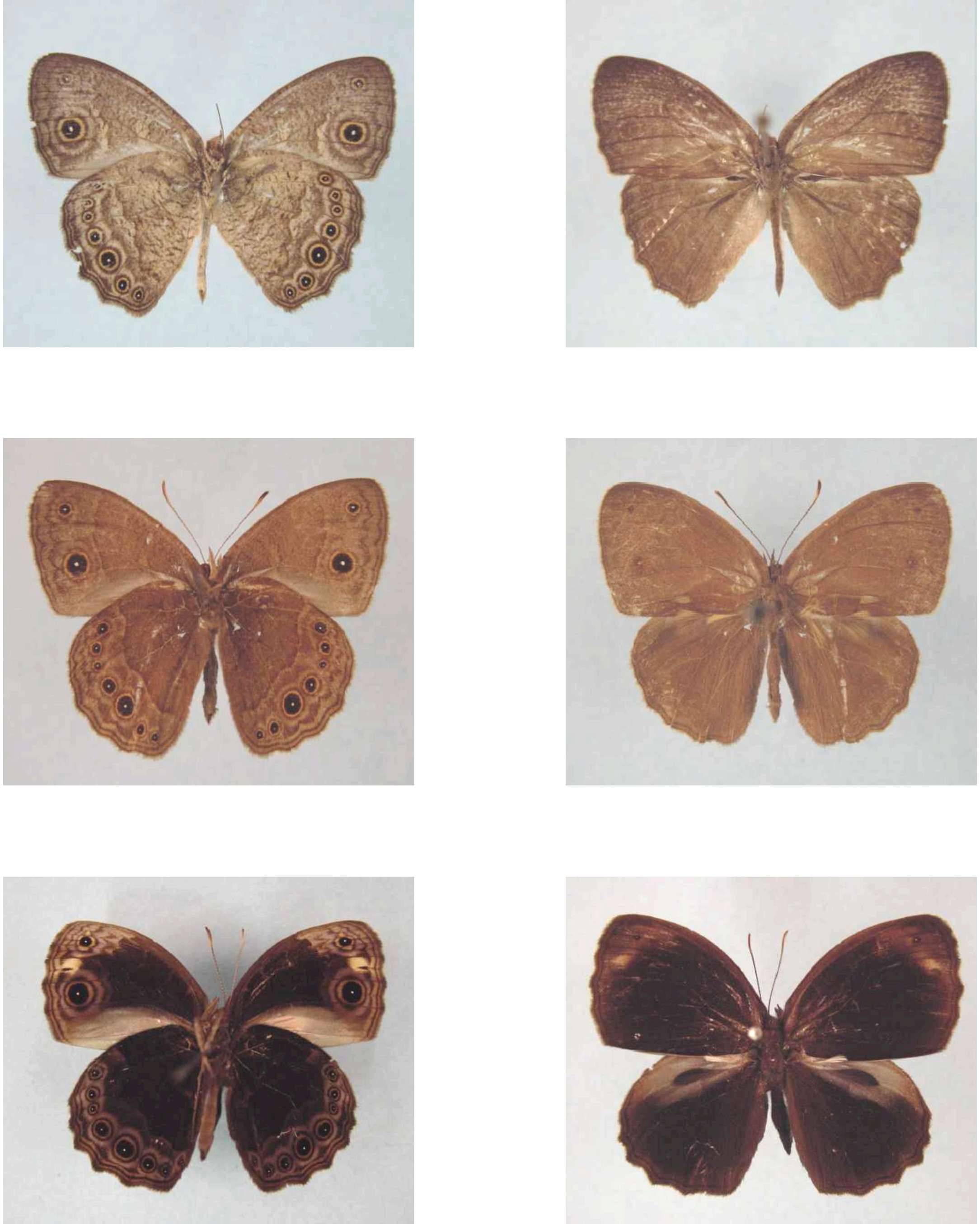

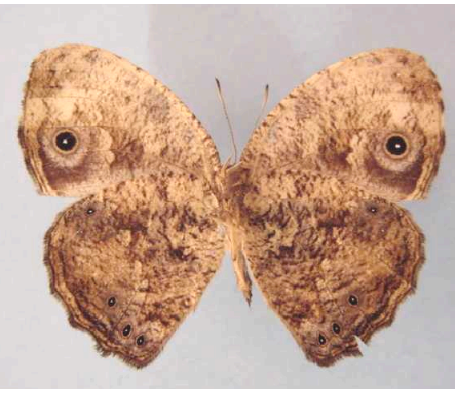

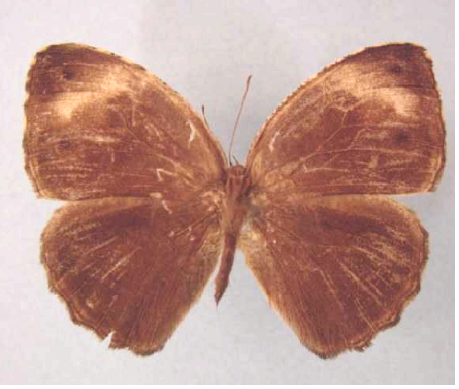

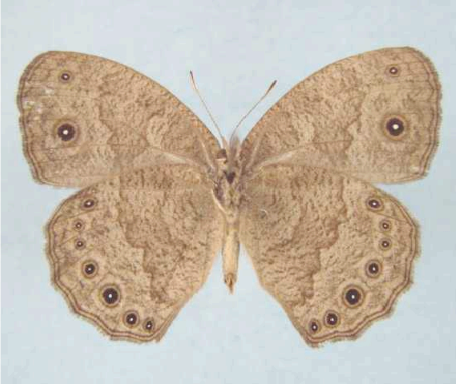

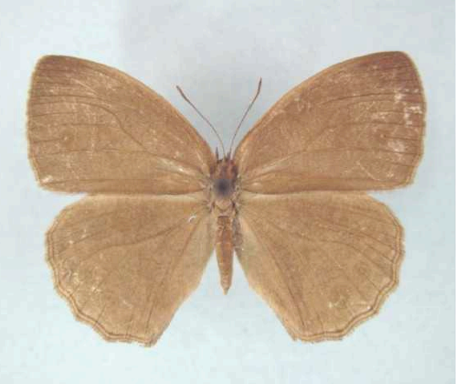

Supplement: S1 Appendix — (PDF) [file pone.0141433.s001.pdf]
