# Supplementary figures and images for: Butterfly Eyespots: Their Potential Influence on Aesthetic Preferences and Conservation Attitudes
Source: PLoS One. 2015 Nov 6;10(11):e0141433. doi: 10.1371/journal.pone.0141433 (PMC4636354; doi:10.1371/journal.pone.0141433)

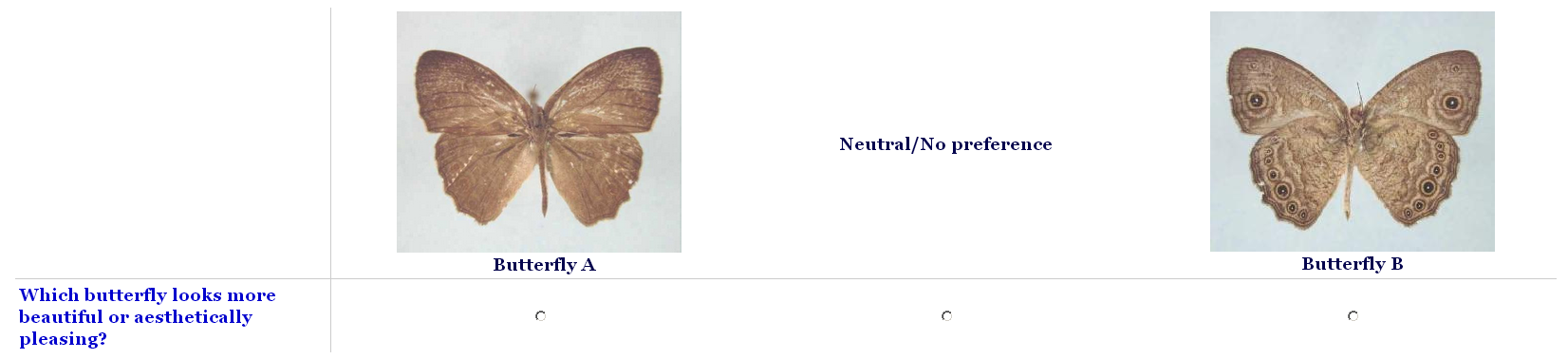

Supplement: S2 Appendix — (TIF) [file pone.0141433.s002.tif]
