# Supplementary material for: Butterfly Eyespots: Their Potential Influence on Aesthetic Preferences and Conservation Attitudes
Source: PLoS One. 2015 Nov 6;10(11):e0141433. doi: 10.1371/journal.pone.0141433 (PMC4636354; doi:10.1371/journal.pone.0141433)
Supplement: S3 Appendix — (PDF) [file pone.0141433.s003.pdf]

Ten-item measure of aesthetic preferences and conservation attitudes (Studies 1 and 4)

Please indicate your preference.

Item*
1. Which butterfly looks more beautiful or aesthetically pleasing?
2. Which butterfly would you like to see more often at your garden (or to attract to your window)?
3. Which butterfly looks stronger or healthier?
4. Which butterfly lives longer and travels longer distances?
5. Which of those butterflies should be protected and maintained to a greater extent?
6. For which of those butterflies should federal government agencies invest more time and resources in butterfly conservation programs?
7. For which of those butterflies should federal governments impose stricter civil penalties for illegal collecting, harassing, or habitat disturbance?
8. For which of those butterflies should the government invest more in research efforts and public education programs?
9. If you were to volunteer in a butterfly conservation program, which butterfly would select to volunteer your time for?
10. If you were to donate some money for butterfly protection, which butterfly would you select to donate your money to?

* Items 1 and 2 constituted the “aesthetic preferences” scale, items 3 and 4 constituted the “perceived fitness” scale and items 5 - 10 constituted the “conservation attitudes” scale.
