# Supplementary material for: Butterfly Eyespots: Their Potential Influence on Aesthetic Preferences and Conservation Attitudes
Source: PLoS One. 2015 Nov 6;10(11):e0141433. doi: 10.1371/journal.pone.0141433 (PMC4636354; doi:10.1371/journal.pone.0141433)

**Butterfly stimuli (Study 4)**

Striped, spotted and spotless *B. anynana* butterflies used in a within-participants design (randomized position).

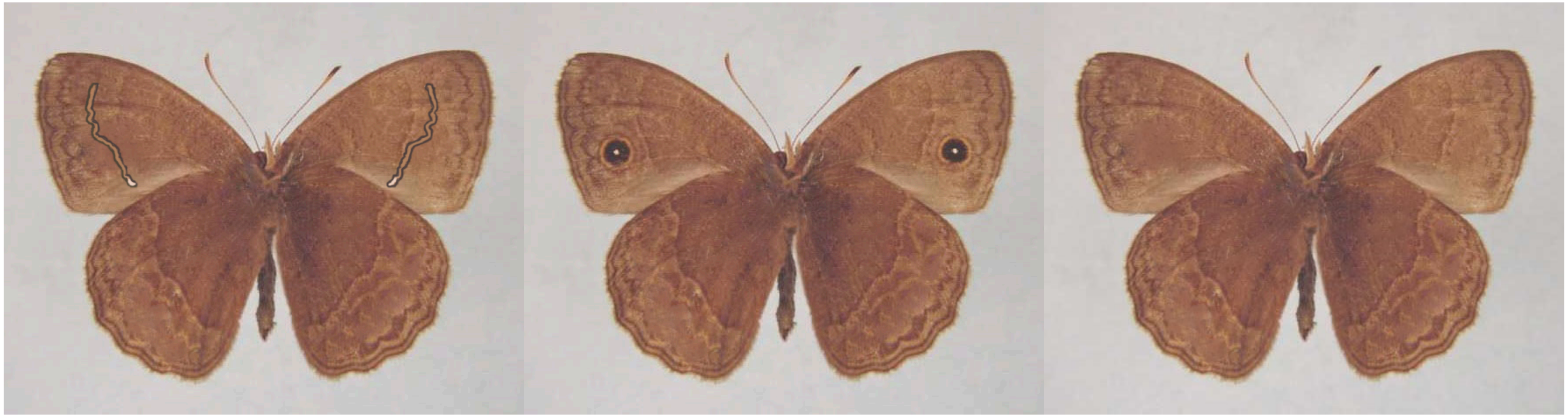

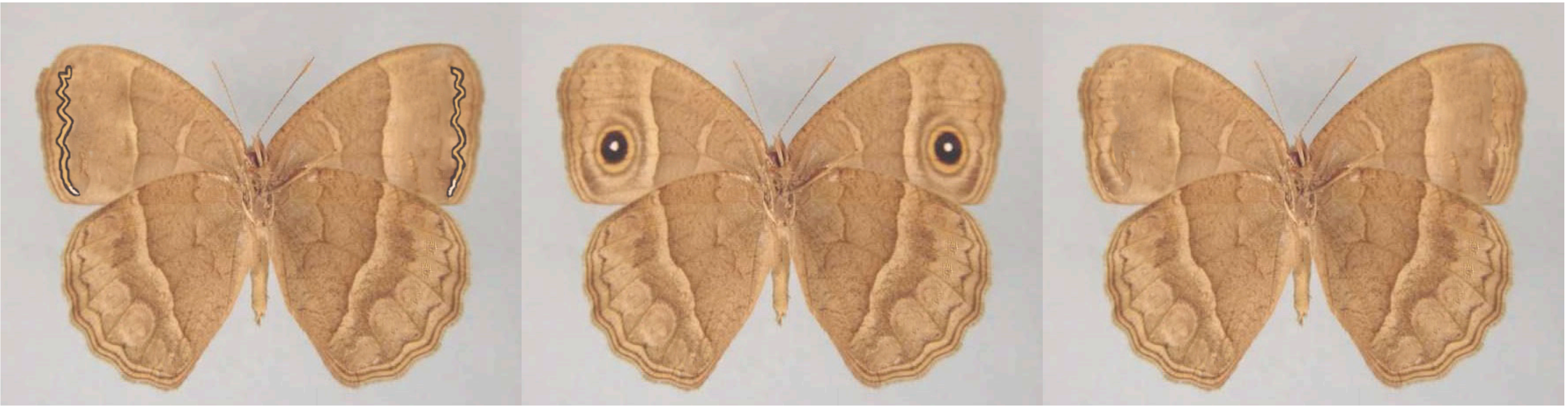

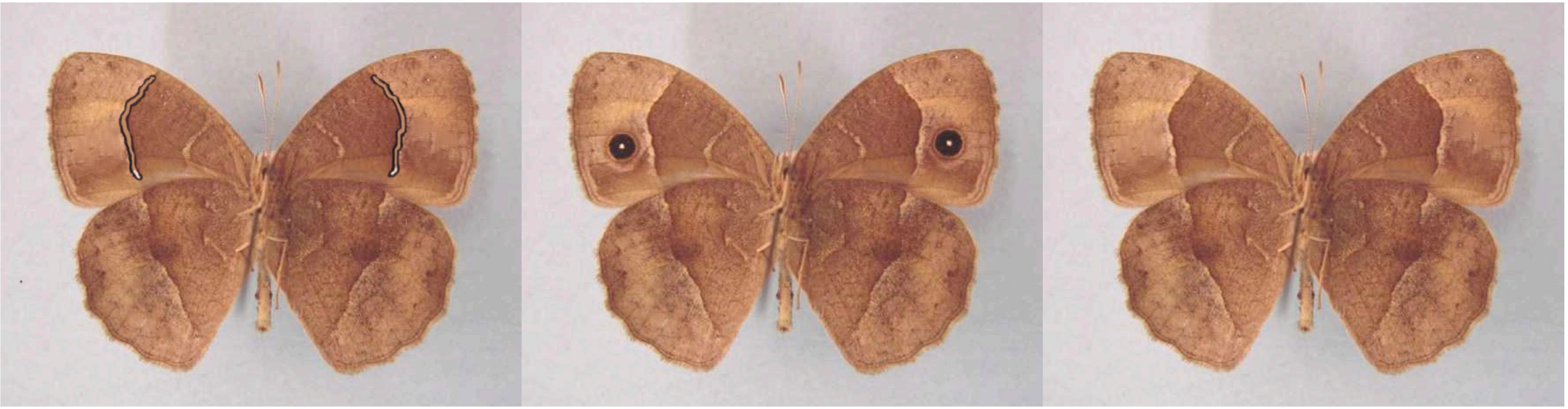

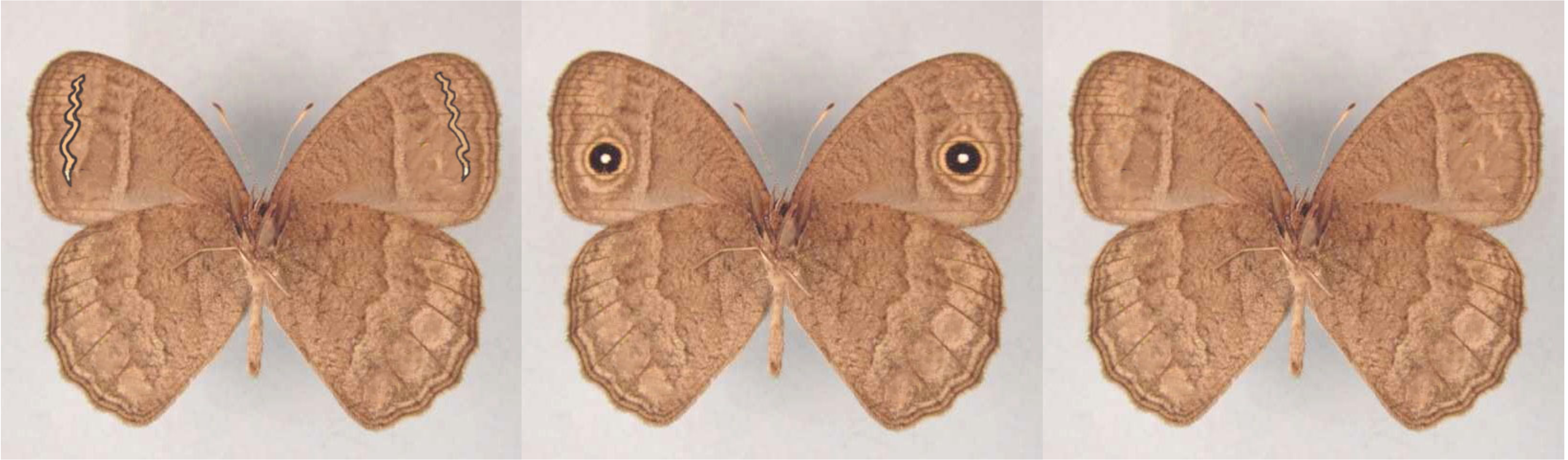

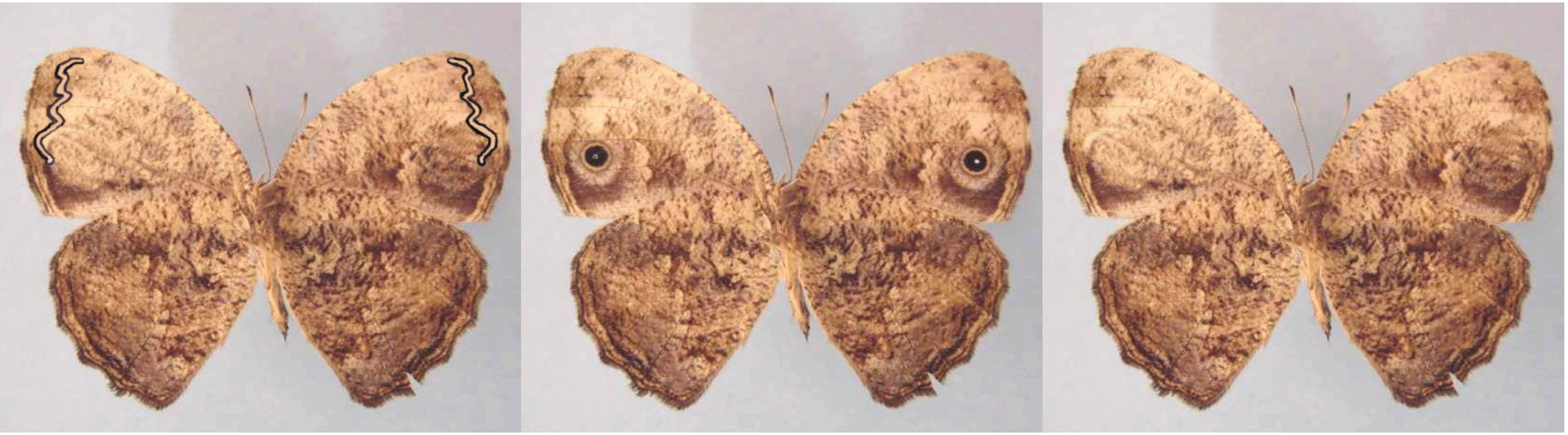

Supplement: S4 Appendix — (ZIP) [file pone.0141433.s004.zip › Appendix S4.pdf]
